# 

**Published:** 1909-06-05

**Authors:** 


### GRADUATES' MEDICAL SCHOOLS.

DIARY FOR THE WEEK, JUNE 7 TO JUNE 12.

### ROYAL SOCIETY OF MEDICINE, 20 Hanover Square, W.

**At 5.30 p.m.**

**June 8, Surgical Section.** Mr. Walter G. Spencer: "Benign Tumours—fibroma, myoma, lipoma—Encapsuled in the Wall of the Stomach."

Mr. E. Stanmore Bishop: "Some Cases of Gastric Surgery."

**At 7.45 p.m.**

**June 10, Obstetrical and Gynæcological Section.** Specimens:—Dr. G. E. Purslow: (1) Uterus, with Fibroid Tumour, from a case in which Cæsarean Hysterectomy was performed. (2) Bilateral Ovarian Tumours, one of which obstructed labour on two occasions.

Dr. W. J. Gow: Cancer Originating Separately in the Uterine Body and in the Cervix.

Short communication.—Mr. J. Bland-Sutton: "Red Degeneration of a Uterine Fibroid associated with the Staphylococcus Pyogenes Aureus."

Mr. G. Darwall Smith: "An Unusual Solid Tumour of the Ovary."

Dr. Kedarnath Das: "Fæcal Chondro-dystrophia."

Papers.—Dr. J. S. Fairbairn: "Primary Chorion Epithelioma of the Ovary."

Dr. Blair Bell: "A Case of Rudimentary Uterus Didelphys, with Ectopia of each Uterine Body in an Inguinal Hernia Sac; with some remarks on the Development of the Female Genital Organs."

### MEDICAL GRADUATES' COLLEGE AND POLYCLINIC, 22 Chenies Street, W.C.

**At 4 p.m.**

**June 7,** Dr. Graham Little, **Skin.**

**At 5.15 p.m.**

**June 7,** Dr. W. H. B. Stoddart, **The Early Signs of Mental Disorder.**

**At 4 p.m.**

**June 8,** Dr. Leonard Guthrie, **Medical.**

**At 5.15 p.m.**

**June 8,** Dr. Hector Mackenzie, **Diagnostic Methods in Tuberculosis.**

**At 4 p.m.**

**June 9.** Mr. E. Laming Evans, **Surgical.**

**At 5.15 p.m.**

**June 9,** Mr. Francis Jaffray, **Appendicitis: Symptoms and Diagnosis.**

**At 4 p.m.**

**June 10,** Sir Jonathan Hutchinson, **Surgical.**

**At 5.15 p.m.**

**June 10,** Mr. Jackson Clarke, **The Clinical Examination of Spinal Cases.**

**At 4 p.m.**

**June 11,** Dr. Dundas Grant, **Ear, Nose, and Throat.**

### THE POST-GRADUATE COLLEGE, West London Hospital, Hammersmith, W.

**At 10 a.m.**

**June 7 and 10,** Surgical Registrar, **Demonstration.**

**June 11,** Medical Registrar, **Demonstration.**

**At 12 noon.**

**June 7,** Dr. Bernstein, **Pathological Demonstration.**

**At 12.15 p.m.**

**June 8 and 9,** Dr. Pritchard, **Practical Medicine.**

**At 5 p.m.**

**June 7,** Mr. Baldwin, **Practical Surgery—II.**

**June 8,** Mr. Etherington Smith, **Clinical Lecture.**

**June 9,** Mr. Pardoe, **Retention of Urine.**

**June 10,** Mr. Keetley, **Clinical Lecture.**

**June 11,** Dr. Abraham, **Cases of Skin Disease.**

### THE THROAT HOSPITAL, Golden Square, W.

**At 5.30 p.m.**

**June 7,** Mr. Faulder, **Diseases of the Perceptive Mechanism.**

**June 10,** Dr. Bond, **Acute and Chronic Inflammation of the Mastoid.**

### NORTH-EAST LONDON POST-GRADUATE COLLEGE, Prince of Wales's General Hospital, Tottenham, N.

**At 4.30 p.m.**

**June 8,** Dr. A. H. Pirie, **The Use of X-rays in Injuries about the Joints.**

**June 10,** Dr. T. R. Whipham, **Demonstration of Cases of Children's Disease.**

### CENTRAL LONDON THROAT AND EAR HOSPITAL, Gray's Inn Road, W.C.

**At 3.45 p.m.**

**June 8,** Mr. Chichele Nourse, **The Middle Ear and Labyrinth.**

### LONDON SCHOOL OF CLINICAL MEDICINE, Seamen's Hospital, Greenwich, S.E.

**At 3.15 p.m.**

**June 7,** Mr. W. Turner, **Accidents Complicating Operations.**

**At 2.15 p.m.**

**June 8,** Dr. Russell Wells, **Failing Heart.**

### NATIONAL HOSPITAL FOR THE PARALYSED AND EPILEPTIC, Queen Sq., Bloomsbury, W.C.

**At 3.30 p.m.**

**June 8 and 11,** Dr. F. Buzzard, **Forms of Paraplegia and their Treatment.**

